# In Vitro Exposure to Glucose Alters the Expression of Phosphorylated Proteins in Platelets

**DOI:** 10.3390/biomedicines11020543

**Published:** 2023-02-13

**Authors:** Mizuho Suzuki, Kyosuke Takeshita, Yuki Kitamura, Marie Kuribayashi, Zhenlie Huang, Gaku Ichihara, Shinji Oikawa, Sahoko Ichihara

**Affiliations:** 1 Department of Environmental and Preventive Medicine, Jichi Medical University School of Medicine, Shimotsuke 329-0498, Japan; 2 Department of Clinical Laboratory, Saitama Medical Center, Saitama University, Saitama 350-8550, Japan; 3 Department of Human Functional Genomics, Life Science Research Center, Mie University, Tsu 514-8507, Japan; 4 Department of Occupational and Environmental Health, Nagoya University Graduate School of Medicine, Nagoya 466-8550, Japan; 5 Department of Environmental and Molecular Medicine, Mie University Graduate School of Medicine, Tsu 514-8507, Japan

**Keywords:** diabetes mellitus, platelet, phosphorylation, hyperactivation, MALDI-TOF/TOF

## Abstract

Diabetes mellitus (DM) is a pro-thrombotic state that can potentially cause serious cardiovascular complications. Platelet hyperactivation plays an important role in these pathological processes, however there is little or no information on the effect of hyperglycemia on platelet proteins. The aim of this study was to identify the molecular targets associated with platelet reactivity under hyperglycemia. Towards this goal, we examined the effects of the exposure of platelets to 1 and 2 h glucose (300 mg/dL) and control (vehicle and osmolality control using mannitol) on platelet proteins (n = 4 samples per group) using two-dimensional fluorescence difference gel electrophoresis (2D-DIGE) combined with MALDI-TOF/TOF tandem mass spectrometry. Two-hour exposure to glucose significantly up-regulated the expression of ATP synthase subunit beta, filamin-A, and L-lactate dehydrogenase A chain in platelets. Pro-Q Diamond staining confirmed the effect of 2 h glucose on vinculin, heat shock protein HSP 90-alpha, filamin-A, and fructose-bisphosphate aldolase A (platelet phosphorylated proteins). The identified proteins are involved in various cellular processes and functions and possibly in platelet reactivity under hyperglycemic conditions.

## 1. Introduction

Type 2 diabetes mellitus (T2DM) is a common disease that accounts for about 90% of diabetes and affects more than 537 million individuals worldwide according to the International Diabetes Federation (IDF) announcement in November 2021 [1]. The number of patients with T2DM continues to increase in countries around the world, including Japan [2]. T2DM can lead to various cardiovascular disorders, such as angiopathies and stroke, which are the major causes of morbidity and mortality in T2DM [3]. It is reported that approximately half of the patients with acute coronary diseases suffer from T2DM [4]. Moreover, the risk of death from any cause among persons with T2DM is up to twice that of the general population, while the risk of death from cardiovascular causes is up to four times that of the general population [5].

It has been shown that patients with DM have a hypercoagulable state due to increased activity of coagulation factors [6,7]. Platelets stimulated with thrombin and collagen form a subpopulation referred to as coated-platelets, which express high levels of surface procoagulant proteins including Factor V, fibrinogen, thrombospondin, alpha-2-antiplasmin, fibronectin, and von Willebrand factor [8]. Coated-platelets also express surface phosphatidylserine and strongly support prothrombinase activity [9]. High levels of these activated platelets have been reported in patients with an acutely ischemic limb and transient ischemic attack (TIA) [10,11]. Moreover, high coated-platelet levels were related to smoking and glucose control drugs in patients with type 1 and 2 DM [12].

A high proportion of activated platelets in the circulating blood and enhanced platelet adhesion are also encountered in patients with T2DM [13]. Platelet activation increases the expression of P-selectin, which facilitates their adhesion onto leukocytes and endothelial cells. Platelets then release physiologically active substances, such as thromboxane A2 (TXA2) and platelet-derived growth factor (PDGF) [14]. TXA2 has a platelet-aggregation action and mediates its effects through thromboxane prostanoid receptors [15]. PDGF induces phenotypic alteration of vascular smooth muscle cells, proliferation, and migration through regulating ROS/NFκB/mTOR/P70S6K signaling pathway [16]. Therefore, long-term platelet activation in the circulating blood of T2DM patients seems to play a major role in the acceleration of arteriosclerosis, diabetic angiopathy, and the onset and progression of thrombosis. Indeed, platelet hyperactivation is involved in the cardiovascular complications associated with T2DM [17].

Reversible phosphorylation of proteins, mainly on tyrosine, threonine, and serine residues, is of great importance among post-translational modifications. Phosphorylation plays an essential role in the regulation of various cellular processes, such as cell cycle, proliferation, apoptosis, and signal pathways that require strict control by protein kinases and protein phosphatases [18]. Protein tyrosine phosphatase 1B (PTP1B), a phosphorylated tyrosine-specific phosphatase, is one of the negative regulators in insulin signal transduction [19]. PTP1B plays a key role in the pathogenesis of T2DM and related complications by dephosphorylating insulin receptors in muscle and liver and attenuating insulin signaling [20]. Platelet hyperactivation in T2DM also involves several other non-platelet-related factors, such as the increased production of reactive oxygen species (ROS), altered Ca^2+^ mobilization, and increased protein tyrosine phosphorylation [21].

Given that platelet reactivity plays an important role in the processes that lead to a hypercoagulable state under hyperglycemic conditions, this study focuses on the effects of glucose on platelet proteins. Towards this goal, we examined the effects of the exposure of platelets to 1 and 2 h glucose (300 mg/dL) and control (vehicle and osmolality control using mannitol) on platelet proteins using two-dimensional fluorescence difference gel electrophoresis (2D-DIGE) combined with MALDI-TOF/TOF tandem mass spectrometry. Hyperglycemia above 300 mg/dL has been shown to be associated with increased mortality, depending on the admission diagnosis [22]. Specifically, plasma glucose levels >300 mg/dL were predictive of a worse prognosis for patients with ST-segment elevation myocardial infarction [23]. Therefore, 300 mg/dL was chosen as the concentration for the glucose exposure. In the present study, we determined the changes in the expression levels of proteins and phosphorylated proteins in platelets exposed to glucose to find possible molecular targets that can explain the mechanisms of platelet hyperreactivity under hyperglycemic conditions.

## 2. Materials and Methods

### 2.1. Sample Treatment

Human platelets prepared from blood for transfusion were purchased from The Japanese Red Cross Society (Tokyo, Japan). The sex of the platelet donor was not disclosed. Platelets were divided into six groups: (1) platelets incubated for 1 or 2 h at 37 °C (n = 4 each), (2) platelets incubated in the presence of 300 mg/dL glucose for 1 or 2 h at 37 °C (n = 4 each), and (3) platelets incubated in the presence of 300 mg/dL mannitol for 1 or 2 h at 37 °C (n = 4 each). The purchased human platelets are suspended in serum. After leaving the platelets, we collected 5 mL of the supernatant serum, added glucose or mannitol to the serum, and returned it to the original platelet solution. Platelets of control groups (1) were incubated without treatment. Platelets of groups (2) and (3) above were incubated under the same osmotic pressure.

### 2.2. Preparation of Platelet-Derived Proteins

The platelets were lysed with 400 μL of 1 × lysis buffer available in the kit (Cell lysis buffer; Cell Signaling Technology, Danvers, MA, USA). After 5 min incubation on ice, the samples were centrifuged for 10 min at 14,000× *g* and 4 °C and the supernatants were removed for use. The supernatant was diluted with the lysis buffer (30 mM Tris-HCl, 7 M urea 2 M thiourea, 4% *w*/*v* CHAPS, and a protease inhibitor cocktail, pH 8.5) and the protein concentration in the supernatant was determined with the BCA protein kit (Thermo Fisher Scientific, Waltham, MA, USA), using bovine serum albumin as a standard [24].

### 2.3. Two-Dimensional Fluorescence Difference Gel Electrophoresis (2D-DIGE)

Each sample was labeled with amine-reactive cyanine dyes, Cy3 or Cy5, developed for fluorescence 2D-DIGE technology (GE Healthcare, Chicago, IL, USA), using the protocol supplied by the manufacturer. Internal pools were generated by combining equal amounts of each sample (n = 4 per group) and labeled with Cy2. Then, two-dimensional gel electrophoresis (2DE) was performed, as described in detail previously [25,26]. After 2DE, cyanin-labeled proteins were visualized directly by scanning using the Typhoon 9400 imager (GE Healthcare) set in fluorescence mode. Image analysis was carried out with DeCyder Differential Analysis Software (DeCyder software V6.0, GE Healthcare), which provides quantification, gel matching, and statistical analysis. The differential in-gel analysis (DIA) module was used for a pairwise comparison of two samples on one gel. For each DIA comparison, the entire signal from each CyDye channel was normalized before the detection of protein-spot boundaries and the calculation of the volume ratio for each protein-spot pair. The biological variation analysis (BVA) module was then used to match all protein-spot maps from comparable gels simultaneously. The average abundance change was calculated from the DIA ratio and the Student’s *t*-test was used for statistical analysis when comparing values of the two groups using DeCyder Software. The differences were confirmed using the Mann–Whitney U test in JMP (JMP 16 software; SAS Institute, Cary, NC, USA). Gel spots with *p* < 0.05 were considered as statistically significant changes.

### 2.4. Protein Identification

After image analysis, the gels containing the additional load of unlabeled proteins from the platelets were stained with Colloidal Coomassie Brilliant Blue G (GE Healthcare) and matched to the fluorescent 2D-DIGE images [26]. Selected spots were picked and in-gel digestion of the protein samples was performed using the protocol described in detail previously [26]. The mass analysis of peptide mixtures was performed using a matrix-assisted laser desorption ionization time-of-flight tandem mass spectrometry (MALDI-TOF/TOF MS; 4800 Plus MALDI TOF/TOF^TM^ Analyzer, Applied Biosystems, Waltham, MA, USA) operating in positive-ion reflector mode. The excised proteins were identified by searching the UniProt protein database by the Paragon Method using the Protein Pilot software V3.0 (Applied Biosystems).

### 2.5. In-Gel Staining of Phosphorylated and Total Proteins

The phosphorylated and total proteins in gels were stained with the Pro-Q Diamond phosphoprotein stain or SYPRO-Ruby protein stain, respectively, following the protocol provided by the manufacturer (Thermo Fisher Scientific) [27]. Protein samples prepared from the control or platelets incubated with glucose for 2 h were separated by 2DE (n = 3 per group). Since the gel staining method for detecting phosphorylated proteins requires a lot of time and effort, only three samples each in the control and loaded-glucose for 2 h were examined. The gels were first stained with Pro-Q Diamond phosphoprotein gel stain to detect phosphoproteins, and visualized using the Typhoon 9400 imager. The gels were then stained with SYPRO-Ruby protein gel stain to reveal the total proteome before being visualized. The analysis process was carried out by matching all gels from the control or glucose-incubated platelets to determine quantitatively the effect of glucose using PDQuest™ 8.0 Advanced 2D Analysis software (Bio-Rad Laboratories, Hercules, CA, USA). The relative abundance of phosphoproteins was calculated in the Pro-Q Diamond images and the SYPRO Ruby images, as described previously [27]. The Mann–Whitney U test was used for statistical analysis when comparing the values of the two groups using the JMP 16 software.

To determine the position of phosphoproteins in the total protein, phosphoprotein spots were matched in gels stained by the Pro-Q Diamond and SYPRO Ruby stains. The matched spots were further matched with the spots in 2D-DIGE gels. Subsequently, phosphoprotein spots detected qualitatively by Pro-Q Diamond staining, combined with SYPRO Ruby staining and also detected quantitatively by 2D-DIGE with a statistically significant difference (*p* < 0.05), were selected. Selected spots were analyzed using MALDI-TOF/TOF MS to identify the proteins, as described above.

### 2.6. UniProt Analysis and Mapping of Protein Expression

Protein ontology classification was performed by importing proteins into the protein analysis using the Universal Protein Resource (UniProt) database (https://www.uniprot.org/; European Bioinformatics Institute, Cambridge, UK: SIM Swiss Institute Bioinformatics, Geneva, Switzerland: Protein Information Resource, Washington, DC, USA) (accessed on 20 December 2021). Proteins that showed changes in their expression levels after incubation with glucose for 2 h were described according to their associated molecular functions, biological processes, and locations.

### 2.7. Statistical Analysis

Data are presented as mean ± standard error of the mean (SEM). Differences between the two groups were tested using the Mann–Whitney U test, and differences among the three groups were tested using one-way analysis of variance (ANOVA) followed by Dunnett’s multiple comparison test. All statistical analyses were conducted using the JMP 16 software. A *p* value < 0.05 was considered statistically significant.

## 3. Results

### 3.1. Glucose Exposure Alters Platelet Protein Expression

Proteins were extracted from human-derived platelets and used for comparative analysis by 2D-DIGE. Image analysis of the gels detected about 2354 spots. The results showed a significant difference in the expression of 63 proteins in the platelets between the control and the loaded-glucose for 1 h, and 50 proteins in the platelets between the control and the loaded-glucose for 2 h by DeCyder software analysis (*p* < 0.05). The results also showed a significant difference in the expression of 34 proteins in the platelets between the control and the loaded-mannitol for 1 h, and 35 proteins in the platelets between the control and the loaded-mannitol for 2 h.

Among the protein spots subjected to MALDI-TOF/MS, seven showed significant changes in the expression of platelets exposed to glucose for 1 h compared with both the control and mannitol (Table 1). The expression levels of spots 282, 700, 715, 895, 1727, and 1734 were significantly up-regulated in 1 h glucose exposed platelets compared with the vehicle and mannitol control (Table 1). On the other hand, the expression level of spot 2129 was significantly down-regulated in the 1 h glucose exposed platelets compared with the vehicle and mannitol control (Table 1). The names of the identified protein are listed in Table 1.

**Figure 1:**
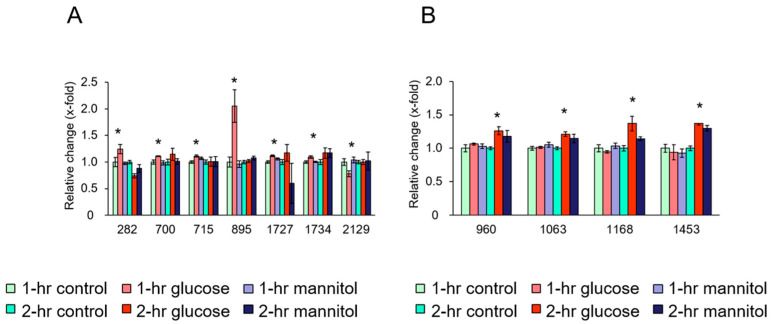
Quantitative effects of glucose on platelet proteins. (**A**) Changes in platelet proteins after 1 h incubation with glucose, osmolality control (mannitol), and vehicle control. (**B**) Changes in platelet proteins after 2 h incubation with glucose, osmolality control (mannitol), and vehicle control. Ordinate: changes in the expression levels measured after and before exposure to the indicated treatment. Abscissa: numerical values represent individual protein spots: (**A**) spot 282: vinculin (VCL), spot 700: calnexin (CANX), spot 715: WD repeat-containing protein 1 (WDR1), spot 895: tubulin beta chain (TUBB), spot 1727: carbonic anhydrase 2 (CA2), spot 1734: Rho GDP-dissociation inhibitor 2 (Rho GDI 2), spot 2129: calpain small subunit 1 (CSS1). (**B**) spot 960: ATP synthase subunit beta (ATP5F1B), spots 1063 and 1168: filamin-A, spot 1453: L-lactate dehydrogenase A chain (LDHA). Data are mean ± SEM of four platelet samples per group. * *p* < 0.05, compared with the corresponding value of the control group.

For platelets exposed to glucose for 2 h, four protein spots showed significantly different expression levels compared with the vehicle and mannitol control (Table 1). The expression levels of spots 960, 1063, 1168, and 1453, indicative of ATP synthase subunit beta (ATP5F1B) (spot 960), filamin-A (FLNA) (spots 1063 and 1168), and L-lactate dehydrogenase A chain (LDHA) (spot 1453), were significantly up-regulated in platelets exposed to glucose for 2 h compared with the vehicle and mannitol control (Table 1). Figure 1A,B show the relative changes in the expression of these platelet proteins in the three groups after 1 and 2 h exposure to glucose.

### 3.2. Glucose Exposure Alters Platelet Phosphorylation Protein Levels

**Figure 2:**
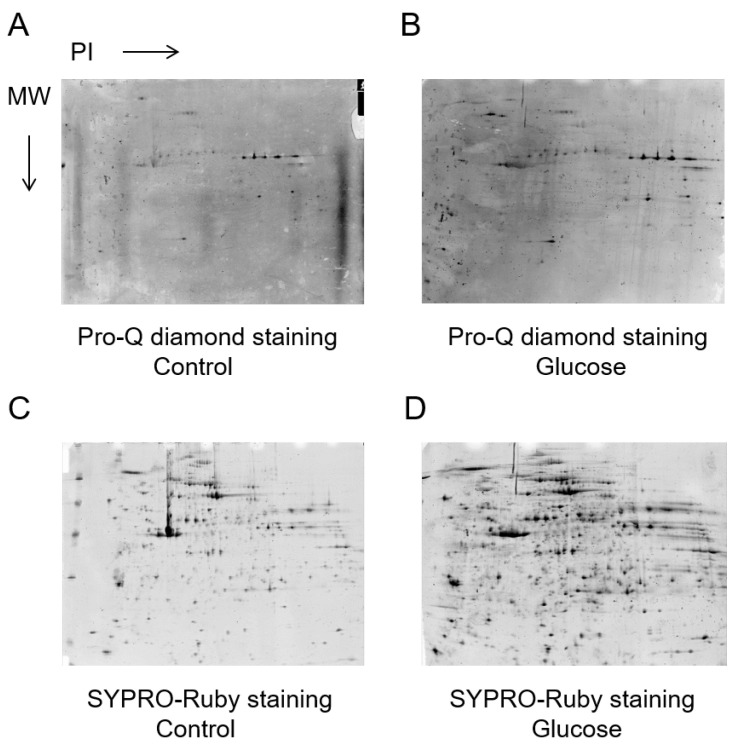
Phosphorylated and total proteins from the same samples were stained in gels with the Pro-Q Diamond and SYPRO-Ruby stain, respectively. Relative Pro-Q Diamond images of platelet proteins of the control (**A**) and 2 h glucose (**B**). Relative SYPRO-Ruby images of platelet proteins of the control (**C**) and 2 h glucose (**D**).

The proteins were extracted from human platelets and subjected to comparative analysis by Pro-Q Diamond staining (Figure 2A,B) and SYPRO Ruby staining (Figure 2C,D). The results showed significant differences in eight platelet phosphorylated proteins between the control and 2 h exposure to glucose, as detected by the PDQuest™ 8.0 Advanced 2D Analysis software (*p* < 0.05).

**Figure 3:**
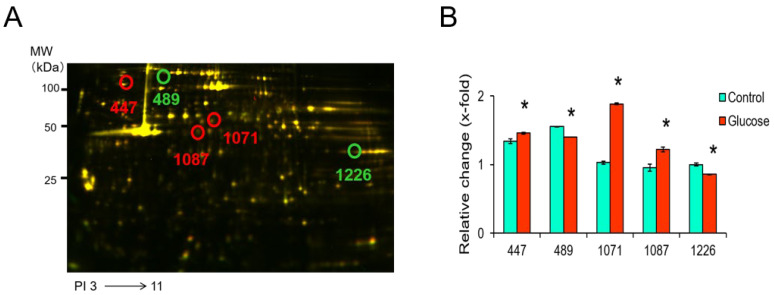
Representative 2D-DIGE image of the platelets treated with vehicle or glucose incubated for 2 h. (**A**) Proteins (40 μg each) were labeled with Cy3 and Cy5 dyes, mixed and subjected to 2D-DIGE analysis. (**B**) Spots 447, 489, 1071, 1087, and 1226 are phosphorylated proteins and their expression was significantly altered by glucose. Data are mean ± SEM of four platelet samples per group. * *p* < 0.05, compared with the corresponding value of the control group using the Mann–Whitney U test.

Figure 3A shows representative 2D-DIGE images of fluorescently labeled proteins in platelets exposed to glucose for 2 h. The peptides mass peaks were compared with those in a protein database using the Paragon Method [28]. The proteins of five spots were identified among eight phosphorylated protein spots, including vinculin (VCL) (spot 447), heat shock protein HSP 90-alpha (HSP90AA1) (spot 489), filamin-A (FLNA) (spots 1071 and 1087), and fructose-bisphosphate aldolase A (ALDOA) (spot 1226) (Table 2). The phosphorylation levels of spots 447, 1071, and 1087 were significantly up-regulated in platelets exposed to glucose for 2 h compared to the control (Figure 3B). On the other hand, the levels of 489 and 1226 were significantly down-regulated in platelets exposed to glucose for 2 h compared with the control (Figure 3B).

### 3.3. Functional Categories of Identified Proteins

Our experiments on the effects of 2 h exposure of platelets to glucose on their proteins and phosphorylated proteins identified a total of six such proteins that exhibited changes in their expression levels. In the next step, these proteins were imported into the UniProt database to understand their functions and locations. Table 3 shows the associated molecular functions, biological processes, and locations of each of these proteins. The molecular functions of these proteins were mainly actin-binding and ATP binding. Furthermore, annotation for the biological processes showed that the altered proteins belonged to cell adhesion, glycolysis, or ATP synthesis. The cellular location varied widely and included the mitochondrion, cytoskeleton, and cytoplasm.

## 4. Discussion

The main finding of our study was the significant effect of glucose on the expression of 50 platelet proteins. In particular, glucose significantly altered the expression of three proteins, with significant up-regulation of the ATP5F1B, FLNA, and LDHA proteins after 2 h exposure. Furthermore, glucose also altered the phosphorylation levels of four platelet proteins, with significant up-regulation of FLNA and VCL and significant down-regulation of the ALDOA and HSP90AA1 proteins after 2 h exposure.

What is the functional importance of these proteins? Our study showed that glucose significantly up-regulated filamin-A and phosphorylated filamin-A in the platelets. Filamins are a family of high molecular mass cytoskeletal proteins that organize actin filaments into networks and link actin networks to the cell membranes [29]. These properties of filamin serve to integrate cell adhesion and signaling systems by providing a scaffold for cytoskeletal proteins and various signaling proteins [30,31]. In mammals, there are three highly homologous filamins: filamins-A, -B, and -C [32]. Among them, filamin-A, encoded by the X-linked gene *FLNA*, is a 280 kDa cytoskeletal protein that cross-links actin in a regulated fashion into either networks or stress fibers [33].

Several studies have shown that filamin-A is related to platelet functions. Jurak et al. [34] demonstrated that filamin-A null megakaryocytes prematurely released large and fragile platelets that were removed rapidly from circulation by macrophages. The degradation of filamin was favorable for the contraction of activated platelets [35]. Moreover, the binding of the platelet GPIb/V/IX (glycoprotein Ib/V/IX) receptor to the von Willebrand factor is critical for platelet adhesion and aggregation under conditions of rapid blood flow [36]. The adhesive function of GPIb*α* is regulated by its anchorage to the membrane skeleton through specific interaction with filamin-A [37]. It has been reported also that filamin-A mutations may lead to impaired or increased α II bβ3 integrin activation, potentially enhancing the risk of thrombosis [33]. In the present study, glucose significantly increased the phosphorylation level of filamin-A in the platelets. Given that filamin-A binding to the cytoplasmic tail of GpIb*α* is involved in platelet activation [38], the result of the present study suggests that the observed increase in phosphorylated filamin-A under hyperglycemic conditions may contribute to platelet hyperactivation and lead to a hypercoagulable state.

The present study also showed that glucose significantly altered various other biological molecules in the platelets. Aldolase A is expressed mainly in muscles and other tissues with a high rate of glycolysis [39]. A number of studies reported that aldolase A interacts with cytoskeletal proteins, especially the actin cytoskeleton, and modulates actin polymerization [40]. Platelets are highly dependent on their actin cytoskeleton for proper functioning. Marked rearrangements of the actin cytoskeleton mediates spreading on matrix proteins and is necessary for thrombus formation. Several actin cytoskeletal regulatory proteins are recruited upon platelet activation [41]. The expression of aldolase has been reported previously to decrease in the platelets of patients with arterial thrombosis or stroke, compared with the healthy subjects [41]. In the present study, glucose significantly reduced the phosphorylation level of aldolase A in the platelets, suggesting that aldolase A is inhibited by the decrease in phosphorylation under hyperglycemia, and this seems to affect platelet function.

Vinculin, a 116 kDa actin-binding protein, is one of the plasma membrane lining proteins that make up the adhesive apparatus of cell adhesion [42]. It mediates integrin binding to the actin filaments of the cytoskeleton and regulates cell adhesion and extension. Mitsios et al. [43] showed previously that megakaryocytes deficient in vinculin exhibited increased membrane tethering in response to mechanical pulling on αIIbβ_3_ with laser tweezers, suggesting that vinculin helps to maintain membrane cytoskeleton integrity. Other studies showed that 95 kDa vinculin fragment binds to the cortical cytoskeletal fraction of lysed platelets in a platelet aggregation-dependent manner, which could be important in the cytoskeletal remodeling of aggregating platelets [44]. Moreover, vinculin has been shown to be a major platelet protein that undergoes Ca^2+^-dependent tyrosine phosphorylation during platelet activation [45]. In the present study, glucose significantly increased the phosphorylation level of vinculin in the platelets, compared with the control, suggesting that such increase may contribute to platelet hyperactivation by causing changes in cytoskeletal organization in the platelets under hyperglycemic conditions.

Previous studies showed that hyperglycemia is associated with increased Rac-1/PAK binding and enhanced Rac-1 translocation from the cytosol to the plasma membrane by ROS [46]. GTPase activity, driven by enhanced Rac activity, leads to actin binding in human platelets [47]. Mechanical strain applied on the actin network is known to activate molecules involved in focal adhesions, such as filamin A and vinculin, and act on integrin [48]. Moreover, the activation of P2Y_12_ in ADP-stimulated platelets enhanced the recruitment of talin to integrin α II bβ3 and activated flaming A [49]. Since platelet P2Y_12_ signaling is activated in patients with diabetes [50], it is possible that under hyperglycemic environment, filamin A, which is activated by the activation of P2Y_12_ signaling, mediates platelet activation in concert with vinculin.

The shotgun proteomics study that investigated the effects of aspirin on platelet protein acetylation reported differences between healthy controls and diabetic patients in proteins of functional pathways, such as several classes of integrin receptors, structural proteins involved in changes in cell morphology (filamin, talin), coagulation factors (V and XIII), and mediators of platelet activation (fibrinogen, P-selection) and degradation (VAMP, SPARC) [51]. Another study that compared protein expression in platelets collected from healthy controls and T2DM patients stored for transfusion found 122 proteins that were either up- or down-regulated in T2DM patients relative to non-diabetic controls [52]. Among these proteins, tubulin beta chain (TUBB), carbonic anhydrase s (CA2), WD repeat-containing protein 1 (WDR1), and calpain small subunit 1 (CSS1) were identified concordantly in the present study, and they were the proteins that changed after 1 h glucose exposure. Although blood glucose level rises after a meal and then falls 1 h after a meal in healthy individuals, it continues to rise up to 2 h after a meal and then gradually falls in diabetics [53]. Based on the results of the above study, we designed our experiment to extract platelet proteins with significant changes in their expression after 1 and 2 h exposure to glucose. Interestingly, there were no common proteins detected in the two time periods. Many studies have shown that postprandial blood glucose levels, i.e., glucose spikes, are more important clinically than the average blood glucose level represented by hemoglobin A1c (HbA1c) with respect to the onset and progression of T2DM [54]. In addition, cohort studies have shown that blood glucose spiking is an independent risk factor for the development of cardiovascular diseases in T2DM [55]. Based on this background, we suggest that the proteins identified in the present study could be involved in platelet reactivity observed in the presence of short-term hyperglycemia rather than under long-term hyperglycemic conditions. Further studies are needed to clarify the molecular mechanisms of these proteins in platelet activation under hyperglycemia.

## 5. Conclusions

Using Pro-Q Diamond staining, we examined the effects of 2 h exposure of peripheral blood platelets to glucose on the level of phosphorylated proteins in these blood components. Glucose exposure altered the expression of four phosphorylated proteins. Our data suggest that phosphorylated proteins, such as filamin A, seem to contribute to platelet reactivity under hyperglycemic conditions. Defective adhesion by blunted interaction with GPIb*α* or defective integrin αIIbβ3 signaling seems a plausible molecular mechanism underlying impaired platelet function under hyperglycemia. Further studies are needed to clarify the roles of the identified proteins in platelet function under hyperglycemic conditions.

## Figures and Tables

**Table 1:** List of proteins with significant spot changes after incubation for 1 and 2 h with glucose or osmolality control (mannitol).

Spot No.	Accession No.	Protein Name	% Cov	Peptides (95%)	Fold Change			* *p*-Value
					Glucose vs. Control	Glucose vs. Mannitol	Mannitol vs. Control	
**1-h exposure**								
282	P18206	Vinculin (VCL)	5.9	3	1.24 *	1.27 *	0.98	0.002
700	P27824	Calnexin (CANX)	10	4	1.11 *	1.13 *	0.99	0.027
715	Q2TAY7	WD repeat-containing protein 1 (WDR1)	17.3	6	1.11 *	1.04	1.07	0.026
895	P07437	Tubulin beta chain (TUBB)	15.5	6	2.05 *	2.14 *	0.96	0.004
1727	P00918	Carbonic anhydrase 2 (CA2)	39.2	8	1.12 *	1.05	1.06	0.024
1734	P52566	Rho GDP-dissociation inhibitor 2 (Rho GDI 2)	33	4	1.09 *	1.09 *	1.00	0.007
2129	P04632	Calpain small subunit 1 (CSS1)	11.2	2	0.78 *	0.75 *	1.04	0.033
								
							
**2-h exposure**								
960	P06576	ATP synthase subunit beta (ATP5F1B)	27.2	9	1.26 *	1.07	1.18	0.027
1063	P21333	Filamin-A (FLNA)	5.4	9	1.21 *	1.06	1.15	0.012
1168	P21333	Filamin-A (FLNA)	3.2	6	1.37 *	1.20	1.14	0.007
1453	P00338	L-lactate dehydrogenase A chain (LDHA)	15.1	4	1.37 *	1.06	1.30	0.014

* *p*-value < 0.05, compared with the corresponding control (ANOVA followed by Dunnett’s multiple comparison test).

**Table 2:** List of the phosphorylated proteins with significant spot changes after 2 h incubation with glucose.

Spot No.	Accession No.	Protein Name	% Cov	Peptides (95%)	Fold Change	* p-Value
447	P18206	Vinculin (VCL)	16.4	9	1.09	0.029
489	P07900	Heat shock protein HSP 90-alpha (HSP90AA1)	2.9	2	0.89	0.029
1071	P21333	Filamin-A (FLNA)	14	8	1.83	0.029
1087	P21333	Filamin-A (FLNA)	12.3	3	1.28	0.030
1226	P04075	Fructose-bisphosphate aldolase A (ALDOA)	27.5	5	0.86	0.030

* *p*-value compared with the corresponding control (Mann–Whitney U test).

**Table 3:** Functional properties and location of platelet proteins that demonstrated a modification in their expression levels due to 2 h glucose exposure.

Protein	Molecular Functions	Biological Processes	Location
**ATP synthesis**			
ATP5F1B	Angiostatin binding, ATP binging, MHC class I protein binding, proton-transporting ATPase activity	Angiogenesis, ATP synthesis, Ion transport	Mitochondrion
**Cell adhesion**			
FLNA	Actin-binding, Actin filament binding, Cadherin binding, Fc-gamma receptor I complex binding, G protein-coupled receptor binding	Actin crosslink formation, Cilium biogenesis/degradation	Cytoskeleton
			
VCL	Actin-binding, Alpha-catenin binding, Beta-catenin binding, Cadherin binding, Dystroglycan binding, Structural molecule activity	Cell Adhesion	Cytoskeleton, Plasma membrane
**Glycolysis**			
LDHA	Oxidoreductase, Hormone activity, Identical protein binding, Thyroid hormone binding	Glycolytic process	Cytoplasm and Cytosol
			
ALDOA	Lyase, Actin binding, Cadherin binding, Cytoskeletal protein binding	Glycolysis	Cytoplasm
**Immune response**			
HSP90AA1	ATP binding, ATP hydrolysis activity, Disordered domain specific binding	Activation of innate immune response	Cytoplasm and Cytosol, Mitochondrion, Nucleus, Plasma membrane

## Data Availability

Data supporting the findings of this study are available within the paper.
